# Hyperuricaemia: a marker of increased cardiovascular risk in rheumatic patients: analysis of the ACT-CVD cohort

**DOI:** 10.1186/1471-2474-15-174

**Published:** 2014-05-23

**Authors:** Inger L Meek, Harald E Vonkeman, Mart AFJ van de Laar

**Affiliations:** 1 Arthritis Centre Twente, University Twente and Medisch Spectrum Twente, Po Box 5000, 7500KA Enschede, Netherlands; 2 UMC St Radboud afd, Reumatische Ziekten, huispost 470, Po Box 9101, 6500HB Nijmegen, Netherlands

**Keywords:** Hyperuricaemia, Gout, Arthritis, Osteoarthritis, Inflammation, Cardiovascular risk, Allopurinol

## Abstract

**Background:**

Gout and hyperuricaemia may be associated with increased cardiovascular risk, but analyses in different populations show conflicting results. This study investigates the impact of serum uric acid, inflammation and traditional CV risk parameters on CV event risk in patients with gouty arthritis and patients with non-gouty rheumatic disease.

**Methods:**

cross-sectional and prospective multivariate analysis of the relation between tertiles of serum uric acid and individual traditional CV risk factors in a cohort of gouty arthritis (GA, n=172), rheumatoid arthritis (RA, n=480) and osteoarthritis (OA, n=206) patients. Main outcome measures: systolic blood pressure, TC/HDL ratio, GlyHb, BMI and first CV events.

**Results:**

Individual CV risk factors were significantly less favourable in GA (systolic blood pressure, TC/HDL ratio, BMI, p<0.05). In RA and OA, but not in GA, individual cardiometabolic parameters correlated with serum uric acid values (OA: RA: systolic blood pressure, TC/HDL ratio, BMI; systolic blood pressure, TC/HDL ratio, GlyHb, BMI; p<0.05). In non-GA individuals the highest tertile of serum uric acid (>0.34 mmol/L) and NT proBNP level were independent predictors of first CV events, against age and GlyHb level in GA (p<0.05). The hazard of first CV events was equally significantly increased in GA patients (HR 3.169, 95% CI 1.287-7.806) and non-GA individuals with a serum uric acid ≥ 0.34 mmol/L (HR 3.721, 95% CI 1.603-8.634) compared to non-GA individuals with a serum uric acid < 0.27.

**Conclusions:**

GA is associated with a 3.1-fold hazard of first CV events. In non-GA rheumatic patients increasing serum uric acid is associated with increased CV risk, whereas CV risk in GA is independent of serum uric acid values. The presence of GA or a baseline serum uric acid in the upper range are possibly stronger predictors of first CV events than some traditional CV risk factors or parameters of inflammation.

## Background

Gouty arthritis (GA) was historically regarded “the king of diseases and the disease of kings”. In modern times GA has become the most prevalent form of inflammatory arthritis and now it is primarily considered a complication of unhealthy Western lifestyles [1,2]. Approximately 5 in every 1000 individuals In European and North American populations suffer from gouty attacks. These individuals also have increased risk for other lifestyle diseases, most notably cardiovascular (CV) events [3].

Gouty inflammation is caused by crystallisation and deposition of uric acid in joints and surrounding tissues. Thus, authors evaluating CV disease in gout have focussed both on hyperuricaemia in a variety of patient populations, and on gouty arthritis (GA) as a clinical entity. These studies show conflicting results. Often hyperuricaemia is found to be an independent risk factor for CV events and death, but in other studies these associations are lost after correcting for traditional CV risk factors. Some studies only find an association with the disease GA [4-17]. There are different pathophysiologic hypotheses that may explain the observed associations: shared risk factors, direct metabolic actions of uric acid on the vascular wall and/or on renin-angiotensin-aldosterone and insulin resistance pathways, or vascular involvement in systemic inflammatory activation. Even though all of these hypotheses are supported by experimental and/or epidemiologic data, none has been definitely confirmed [18,19]. Causality in gout associated cardiovascular risk thus remains unelucidated and pathways are probably complex.

Studies that evaluate the associations between serum uric acid, inflammation and CV risk in rheumatic disease are scarce [20,21]. We therefore investigated the associations between serum uric acid and CV risk parameters and first CV events in patients with different rheumatic diseases. To explore the value of serum uric acid level as a marker of future CV event risk in rheumatic patients a prospective multivariate analysis in GA and non-GA individuals was performed.

## Methods

Data for this study were obtained from the Arthritis Center Twente CardioVascular Disease (ACT-CVD) database. In 2009 the Arthritis Center Twente in Enschede, the Netherlands, established a per protocol cardiovascular screening as standard care, which details have been described previously [22]. Both existing and new patients are screened for traditional CV risk factors and followed for the occurrence of CV events. Briefly, the ACT-CVD database is a collection of the routine clinical care parameters obtained at the initial screening (demographics, traditional CV risk factors, inflammatory parameters, rheumatic disease characteristics and medication), as well as CV event follow up data for each patient. Patients are classified according to their clinical diagnosis as registered by their attending rheumatologist. After screening, each patient is followed for the occurrence of CV events or death. CV events are defined as (1) myocardial infarction; (2) coronary intervention, i.e. percutaneous transluminal coronary angioplasty (PTCA) or coronary artery bypass graft (CABG); (3) angina pectoris, confirmed by a cardiologist as cardiac chest pain; (4) acute heart failure; (5) cerebral vascular accident (CVA); (6) death due to cardiac causes; (7) sudden death. Follow up data are extracted from the hospital electronic registration system and subsequently validated by medical chart review. Out of hospital events and death are documented by periodic questionnaires sent to attending general practitioners and by review of the Dutch national registry of death certificates. For this study the data of all patients without prior documented CV event and with a diagnosis of either rheumatoid arthritis (RA), osteoarthritis (OA) or gouty arthritis (GA) that were screened between February 2009 and December 2011 were selected. Patient follow up ended at December 2012.

In the analysis we used the following definitions: hyperuricaemia: serum uric acid above 0.36 mmol/l in women or 0.40 mmol/l in men; uric acid lowering therapy (ULT): two or more consecutive prescriptions of allopurinol and/or benzbromarone covering at least a 3 months interval; antihypertensive therapy: the use of beta-blocking agents, calcium antagonists, angiotensin converting enzyme inhibitors, angiotensin receptor inhibitors and/or diuretic registered for use as an antihypertensive agent; smoking: the current use of inhaled tobacco; diabetes: the use of glucose lowering medication and/or a fasting plasma glucose above 6.9 mmol/l. Glomerular filtration rate (GFR) as a parameter of renal function was estimated using the ‘modification of diet in renal disease’ (MDRD) formula [23]. Body mass index (BMI) was calculated as the ratio between weight and the square of length.

To evaluate patterns in associations between uric acid and CV risk parameters among rheumatic patients and to define homogeneous groups for the prospective analysis a first cross sectional analysis was performed of the relation between tertiles of serum uric acid and the baseline traditional CV risk parameters systolic blood pressure, total cholesterol/high density lipoprotein (TC/HDL) ratio, glycated haemoglobin (GlyHb) and BMI in patients with individual rheumatic diagnoses. In the prospective COX-regression analysis the predictive value of uric acid tertiles for the occurrence of first CV events in the ACT-CVD population was evaluated considering all abovementioned traditional CV risk parameters and the variables age, sex, high sensitivity CRP (hsCRP), N-terminal pro-brain natriuretic peptide (NT-proBNP), estimated GFR, use of antihypertensive therapy or statins, and in GA patients use of allopurinol or ULT in general. For the prospective analysis duration of follow up was calculated as the interval between inclusion into the cohort and the occurrence of a first CV event or death, or censored at December 1st 2012, whichever came first.

The protocol for data collection, storage, and use in the present study was approved by the Arthritis Center Twente Institutional Review Board. Because the study uses data collected as part of daily clinical care the ethics committees determined, in accordance to Dutch law, that no approval was required. Nonetheless, patients were fully informed and only the data of patients that gave informed consent were entered into the ACT-CVD database.

### Statistical analysis

Prevalence of CV risk factors in patient groups and 10-year CV risk estimates were presented by descriptive statistics (mean or percentage prevalence). Differences between groups and associations between CV risk variables and tertiles of serum uric acid were tested with ANOVA (for continuous CV risk factors) or Chi squared statistics (for nominal CV risk factors), adjusted for differences by age and sex. For the survival analysis those groups that showed similar baseline patterns of CV risk parameters in tertiles of serum uric acid and equal occurrence of first CV events were combined. Kaplan Meier curves were made for CV event free survival over time and backwards stepwise COX regression analysis was performed to determine the value of tertiles of uric acid as an independent predictor for CV event risk over time. Data analysis was performed using PASW Statistics version 18.0.

## Results

After the first year of screening the completed data of 973 individuals with GA (n = 204), RA (n = 533), or OA (222) were available for analysis. Of these individuals 133 (32 GA, 38 RA, 14 OA) were excluded because of prior CV events. Prior CV events were significantly more frequent in GA patients (p < 0.05).

### Baseline CV risk

The baseline characteristics of the study population are presented in Table 1. Distributions of age and sex were as expected. Individuals were predominantly late middle aged, with overrepresentation of women in RA and OA, and men in GA. The hsCRP level, a measure of systemic inflammation, was significantly lower in OA.

**Table 1:** Baseline characteristics

	**GA (172)**	**RA (480)**	**OA (206)**
Serum uric acid, mean, mmol/l (SD)	0.36 (0.14)	0.31 (0.075)**	0.31 (0.0832)**
Hyperuricaemia, n (%)	54 (32)^†^	62 (14)^†^	34 (17)^†^
Uric acid lowering therapy, n (%)	126 (73)	0	0
Allopurinol, n (%)	111 (65)	0	0
Sex, n (%) male	154 (89)	133 (28)**	43 (21)**
Age, mean, years (SD)	59.6 (10.8)	59.0 (13.0)	59.2 (11.0)
Systolic blood pressure, mean, mmHg (SD)	151.0 (21.3)	144.0 (22.9)**	145.5 (20.2)**
Antihypertensive therapy, n (%)	67 (39.0)	118 (24.6)	71 (34.5)
Smoking, n (%)	43 (25)	114 (24)	38 (18)
TC/HDL ratio, mean (SD)	4.82 (1.4)^†^	3.65 (1.1)^†^	4.0 (1.2)^†^
Total cholesterol, mean (SD)	5.32 (1.1)	5.30 (1.0)	5.57 (1.1)
HDL cholesterol, mean (SD)	1.17 (0.32)	1.53 (0.43)	1.48 (0.39)
Statin therapy, n (%)	22 (12.8)	32 (6.7)^‡^	26 (12.6)
Diabetes, n (%)	11 (6.4)	28 (5.8)	16 (7.8)
GlyHb, mean, % Hb (SD)	6.1 (1.4)	5.5 (1.8)	6.0 (4.2)
BMI, mean, kg/m^2^ (SD)	30.4 (4.7)^†^	27.0 (4.3)^†^	28.8 (5.3)^†^
MDRD, mean, ml/min (SD)	80.8 (21.8)	89.9 (19.5)**	87.7 (18.7)**
NT proBNP, mean, nmol/L (SD)	37.6 (170.6)	15.9 (25.8)**	15.1 (32.8)**
hsCRP, mean, g/L (SD)	6.3 (11.2)	7.0 (10.0)	3.8 (4.9)*
Corticosteroids, n (%)	0 (0.0)	68 (14.2)^¥^	1 (0.50)

GA: gouty arthritis; OA: osteoarthritis; RA: rheumatoid arthritis; hsCRP: high sensitivity C-reactive protein; SD: standard deviation; CV: cardiovascular; TC: Total cholesterol; HDL: high density lipoprotein; GlyHb: glycated haemoglobin; BMI: body mass index; MDRD: modification of diet in renal disease; NT proBNP: N-terminal pro-brain natriuretic peptide. *p < 0.05 OA vs. RA and GA, **p < 0.05 RA and OA vs. GA, ^†^p < 0.05 RA vs. OA vs. GA, ^‡^p < 0.05 RA vs. OA and GA., ^¥^p < 0.05 RA vs. GA and OA.

**Figure 1:**
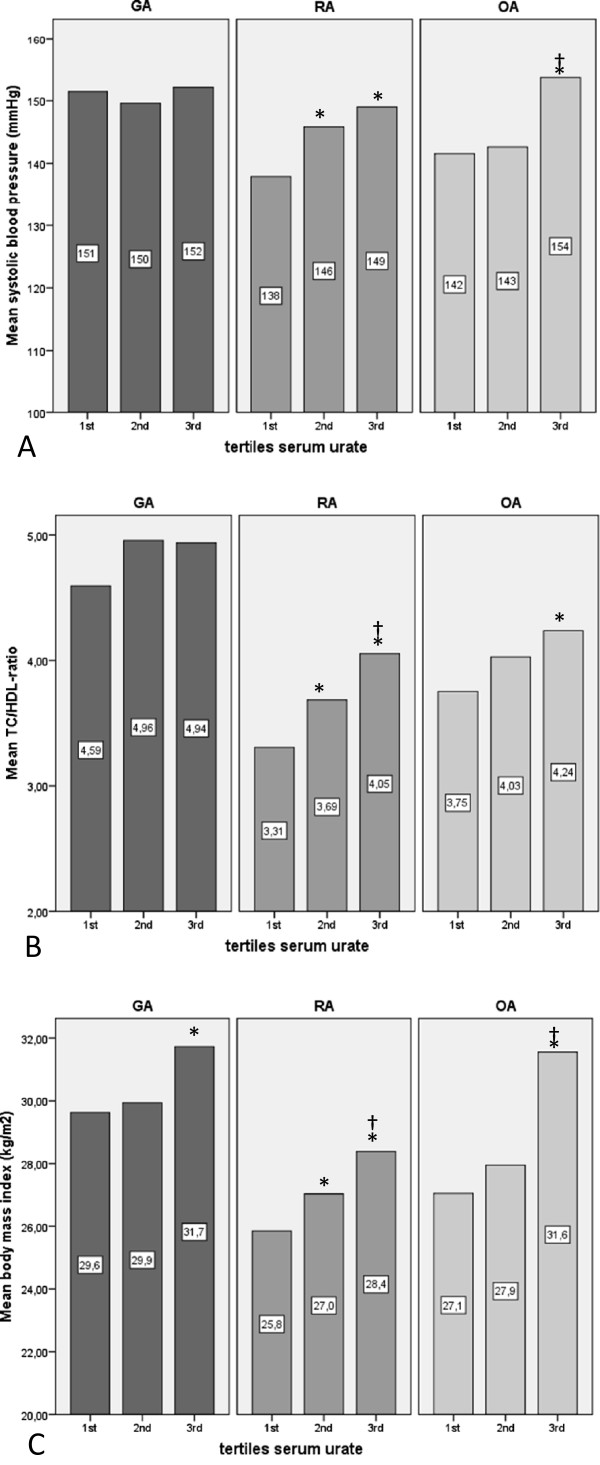
**Associations between baseline cardiovascular risk parameters (A-C) and tertiles serum uric acid in GA, RA and OA.** Tertiles serum uric acid (1^st^, 2^nd^, 3^rd^): GA: <0.29 mmol/L, 0.29-0.38 mmol/L, and ≥0.39 mmol/L; RA: <0.27 mmol/L, 0.27-0.34 mmol/L, and ≥0.34 mmol/L; OA <0.27 mmol/L, 0.27-0.34 mmol/L, and ≥0.34 mmol/L. GA: gouty arthritis; RA: rheumatoid arthritis; OA: osteoarthritis; TC: total cholesterol; HDL: high density lipoprotein; BMI: body mass index. (*p < 0.05 vs. 1^st^ tertile; ^‡^p < 0.05 vs. 2^nd^ tertile).

At baseline the distributions of the traditional CV risk parameters male sex, systolic blood pressure, TC/HDL ratio, and BMI were significantly less favourable in GA patients (p < 0.05). In RA and OA the individual CV risk parameters systolic blood pressure, TC/HDL ratio and BMI, and in OA also GlyHb, all correlated with serum uric acid levels (Figure 1). In GA no associations between serum uric acid and individual CV risk parameters were observed.

The majority of gout patients (73%) used ULT (88% allopurinol, 12% benzbromarone). Mean serum uric acid was significantly lower in patients using ULT (0.32 mmol/l ULT vs. 0.48 mmol/l Non-ULT, p < 0.05). Patients treated with allopurinol had significantly lower hsCRP (14.1 ± 22.6 vs. 4.40 ± 4.41 g/L) and NT-proBNP (76.7 ± 313.7 vs. 24.0 ± 70.2 pmol/L) levels than non-allopurinol treated patients. Otherwise, these patients did not differ in baseline measurements of traditional CV risk factors, or in frequency of treatment with statin or antihypertensive therapy.

### Prospective analysis of CV events

After a median follow up of 36 months (25^th^- 75^th^ percentile 30–41) 64 CV events had occurred, 29 (6.0%) RA, 17 (8.3%) OA, and 18 (10.5%) GA. Five of these first CV events were fatal, 2/29 (6.9%) in the RA, 0/17 (0.0%) in the OA group, and 3/18 (16.6%) in the GA group. Because baseline patterns of traditional CV risk factors in tertiles of uric acid were similar and the occurrence of CV events did not differ significantly between RA and OA patients, the data of RA and OA groups were combined as ‘non-GA’ for further survival analysis. Table 2 shows the univariate associations between baseline variables and occurrence of CV events in non-GA and GA groups.

**Table 2:** Univariate associations between CV risk variables and prospective CV events in GA and non-GA patients

	**GA (172)**			**Non-GA(686)**		
	**Event-free (154)**	**Event (18)**	***p (event vs. none)***	**Event-free (640)**	**Event (46)**	***p (event vs. none)***
Tertiles serum uric acid (% 1^st^/2^nd^/3^rd^)	37.3/32.0/30.7	33.3/16.7/50.0	0.208	40.6/28.3/31.2	15.9/25.0/59.1	0.000*
Uric acid lowering therapy, %	74.0	66.7	0.504	0	0	n.a.
Allopurinol, %	64.3	66.7	0.842	0	0	n.a.
Sex, % male	91.6	72.2	0.011*	24.8	37.0	0.069*
Age, mean, years	58.5	68.8	0.000*	58.6	64.9	0.001*
Systolic blood pressure, mean, mmHg	152.0	143.0	0.092*	143.9	152.1	0.015*
Antihypertensive therapy, %	34.4	77.8	0.000*	26.3	45.7	0.004*
Smoking, %	26.0	11.1	0.147*	22.0	23.9	0.767
TC/HDL ratio, mean	4.83	4.71	0.623	3.74	3.95	0.265
Total cholesterol, mean	5.38	4.83	0.037*	5.38	5.19	0.234
HDL cholesterol, mean	1.18	1.05	0.104*	1.53	1.40	0.057*
Statin therapy, %	11.7	22.2	0.205	8.0	15.2	0.088*
Diabetes, %	5.8	11.1	0.387	6.4	6.5	0.975
GlyHb, mean, %Hb	5.83	6.58	0.004*	5.83	5.91	0.408
BMI, mean, kg/m^2^	30.1	32.9	0.017*	27.4	28.8	0.058*
MDRD, mean, ml/min	83.5	58.1	0.002*	89.7	83.2	0.091*
NT proBNP, mean, nmol/L	14.5	230.9	0.083*	14.1	45.4	0.005*
hsCRP, mean, g/l	5.76	9.44	0.238	5.90	7.21	0.390
Corticosteroids, %	0.0	0.0	-	10.0	10.9	0.850

GA: gouty arthritis; hsCRP: high sensitivity C-reactive protein; TC: Total cholesterol; HDL: high density lipoprotein; GlyHb: glycated haemoglobin; BMI: body mass index; MDRD: modification of diet in renal disease; NT proBNP: N-terminal pro-brain natriuretic peptide. *variable included in the respective COX regression analyses.

In female GA patients the frequency of CV events was unexpectedly high: 5/18 (28%) vs. 13/154 (8.4%) in male GA patients, 29/510 (5.7%) in female non-GA patients and 17/176 (9.7%) in male non-GA patients. Female GA patients were significantly older than male GA patients (female GA mean 70 years ± 10.2 vs. male GA 58 years ± 10.2, p < 0.05), whereas female and male non-GA patients were of comparable age (female non-GA mean 58 years ± 12.6 vs. male GA 58 years ± 12.0, p = 0.465).

**Figure 2:**
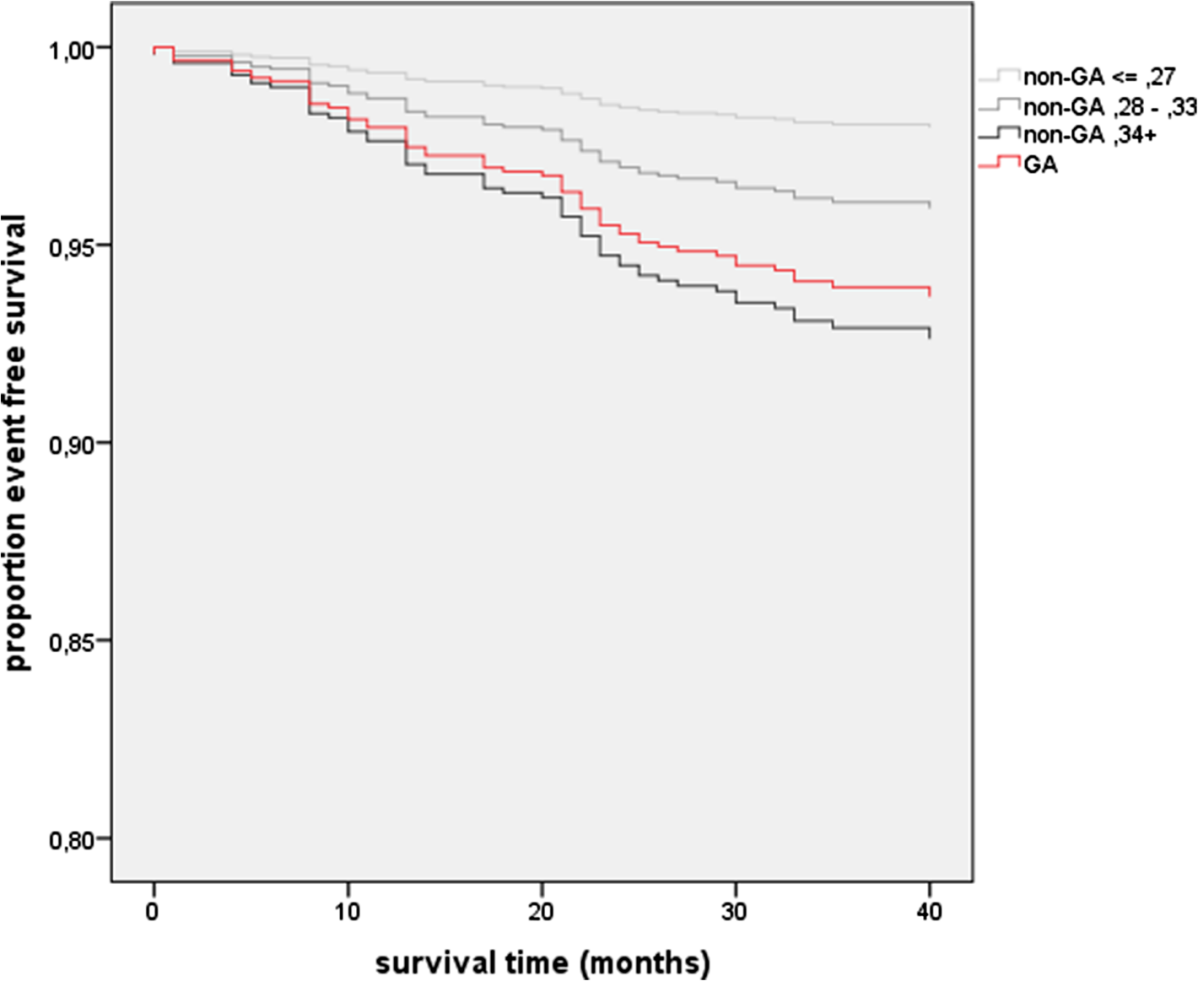
**Comparison of cardiovascular event-free survival in GA and non-GA patients.** Comparison of cardiovascular event-free survival in GA patients and in tertiles serum uric acid (<0.27 mmol/L, 0.27-0.34 mmol/L, and ≥0.34 mmol/L) in non-GA patients. GA: gouty arthritis.

For both GA and non-GA groups all variables with p ≤ 0.150 were included in the backwards stepwise COX regression analyses. The use of uric acid lowering, antihypertensive and/or statin therapy were considered as possible confounders. In the non-GA group the highest tertile of serum uric acid (≥0.34 mmol/L; HR 3.896, 95% CI 1.677-9.051) and NT proBNP (HR 1.012, 95% CI 1.008-1.016) level remained as independent predictors of CV events, against age (HR 1.073, 95% CI 1.022-1.127) and GlyHb level (HR 3.273, 95% CI 1.971-5.434) in the GA group (p < 0.05). Compared to non-GA individuals with a serum uric acid < 0.27, non-GA individuals with a serum uric acid ≥ 0.34 mmol/L (HR 3.721, 95% CI 1.603-8.634) and GA patients (HR 3.169, 95% CI 1.287-7.806) showed equally increased hazard ratio’s for first CV events (Figure 2).

## Discussion

Our data show that GA is associated with a more unfavourable traditional CV risk profile and a 3.1-fold hazard of first CV events compared to non-GA individuals with normal serum uric acid. In non-GA individuals increasing serum uric acid is associated with increased CV risk, the 3.7 fold hazard of a first CV event in non-GA individuals with a serum uric acid ≥ 0.34 mmol/L being comparable with that of gout patients.

Individual traditional CV risk factors, such as systolic blood pressure, serum lipoproteins, and body mass index, and occurrence of first CV events were increased at the same level in subsequent tertiles of serum uric acid in both RA and OA. These associations thus seem relatively independent from chronic inflammation. Compared to the other rheumatic populations mean serum uric acid values were highest in GA, as were values of traditional CV risk factors. First CV events were also more frequent in GA individuals than in non-GA patients. The frequency of first CV events was particularly high in female GA patients. This may be explained by the age of female GA patients, which was 10 years increased compared to the other patient groups, increasing age beyond the menopause being an important CV risk factor in women. The relatively high age of the female GA patients included in the ACT-CVD cohort is in line with previously published epidemiologic studies [24]. Another explanation could be selection bias due to the small number of female GA patients included.

Individual CV risk parameters and prospective CV events were not related to tertiles of serum uric acid in GA patients. This may be due to confounding by the use of uric acid lowering therapy or more specifically the xantine oxidase inhibitor allopurinol, or to a ceiling effect in the generally high risk GA patients. CV risk factor values were not lower in GA patients treated with UTL or specifically allopurinol, and CV events were as frequent. The only suggestion in our data that treatment with allopurinol may be beneficial was the significantly lower mean level of NT proBNP, a marker of cardiac dysfunction, in this group.

### Comparison to the literature

Previously, several large studies found increased CV disease and mortality in patients with gout, approximately two-fold compared to the general population [25-27]. To determine if hyperuricaemia per se confers the same risk of increased CV disease different populations have been studied; the general population, young and elderly, diabetics and patients with chronic kidney disease [4,6-17,28-30]. Many studies did find hyperuricaemia to be an independent risk factor, but in others the association disappeared after correction for the traditional CV risk factors obesity, hypertension, dislipidemia and/or insulin resistance. Some studies found a U- or J-shaped association between serum uric acid level and mortality, suggesting an optimal level between 0.30 and 0.41 mmol/L [29-31]. Only one study specifically addressed the role of systemic inflammation in GA related CV disease, and found no association with CV events [7].

The question remains if serum uric is only a potentially useful marker to improve the selection of high CV risk individuals for CV risk management, or if it is causally related to the progression of CV disease. Different hypotheses have been suggested: 1) the presence of shared risk factors in CV disease and GA, 2) vascular wall activation and accelerated atherosclerosis by chronic systemic inflammation, or 3) a direct interaction of uric acid with diverse metabolic pathways involved in CV disease [18,19]. Several experimental studies have provided insight into possible actions of uric acid in diverse metabolic pathways, i.e. glucose and lipoprotein handling, nitric oxide metabolism, the renin-angiotensin-aldosterone system (RAAS), and inflammatory signalling and activation [32-34]. These observations suggest that uric acid itself can at least modulate CV risk factors such as blood pressure, cholesterol, diabetes and chronic inflammation [35]. Uric acid clearance may be decreased in states of hypertension and RAAS activation, with consequent increasing hyperuricaemia and amplification of metabolic derangements. However, other studies suggest that the metabolic effects attributed to uric acid are actually mediated by xantine oxidase, which is also the key enzyme in uric acid production in man. Increased serum uric acid may thus only be an epiphenomenon of other pathologic metabolic pathways that cause increased CV risk [36].

### Strengths and limitations

Our study adds to the knowledge on hyperuricaemia and CV risk by comparing the association between serum uric acid and CV-risk in a mixed rheumatic population including GA. Thus we saw that in this population either the presence of GA or a baseline serum uric acid in the upper range are stronger predictors of first CV events than some traditional risk factors or parameters of inflammation. Epidemiologic studies that evaluate associations of serum uric acid with traditional CV risk factors and prospective CV events in a rheumatic population are scarce. In this study the study groups were well defined and measurements of risk factors were performed within the same protocol.

However, this study has some important limitations. First, the observational study design precludes any suggestions on causal relationships. Thus the absence of an association between serum uric acid and CV risk in GA was difficult to interpret due to the pre-treated population. Because we only had data on baseline variables we could not evaluate of the effect of medical interventions and changes in serum uric acid on CV outcome. A possible risk reduction by allopurinol may not have followed from our data due to insufficient numbers of patients, too short period of exposure or too short follow up. Following the data one can only conclude that also in a ULT treated GA population with satisfactory mean uric acid levels CV risk is high.

The prospective analysis, evaluating the associations of a limited set of traditional CV risk factors and serum uric acid tertiles in a mixed population of articular diseases, is a simplification that may ignore any disease specific CV risk factors. Moreover, the rheumatic diseases studied differ importantly in general gender epidemiology, reflected by a male predominance in the GA group versus a female predominance in the non-GA group. Gender is an important traditional CV risk factor. Therefore, gender, diagnosis and hsCRP were considered as variables in the multivariate analysis, but proved non-significant. The influence of some other potential confounders such as use of NSAIDs and relative physical inactivity was not covered by separate variables, but regarded to be limited by only including patients with rheumatic diseases.

Some important questions remain for further research. First, the predictive value of pre-ULT treatment uric acid level for the occurrence of CV events in GA patients. Second, the positioning of uric acid and xantine oxidase in pathophysiologic pathways that cause CV events in GA and hyperuricaemia. And finally, what is the optimal risk intervention strategy in high CV risk patients characterised by an upper range uric acid level.

## Conclusions

Both gouty arthritis and, in non-gouty rheumatic patients, upper range serum uric acid are associated with an approximately 3-fold hazard of first CV events. CV risk in gouty arthritis is independent of serum uric acid values and remains important in patients treated to satisfactory uric acid values by uric acid lowering therapy. The presence of a diagnosis of gouty arthritis or a baseline serum uric acid in the upper range are possibly stronger predictors of first CV events than some traditional CV risk factors or parameters of inflammation.

## Abbreviations

GA: Gouty arthritis; OA: Osteoarthritis; RA: Rheumatoid arthritis; TC: Total cholesterol; HDL: High density lipoprotein; GFR: Glomerular filtration rate; MDRD: Modification of diet in renal disease; BMI: Body mass index; ULT: Uric acid lowering therapy; hsCRP: High sensitivity C-reactive protein; NT-proBNP: N-terminal pro-brain natriuretic peptide; HR: Hazard ratio; CI: Confidence interval; RAAS: Renin-angiotensin-aldosterone system.

## Competing interests

All authors have completed the Unified Competing Interest form at http://www.icmje.org/coi_disclosure.pdf (available on request from the corresponding author) and declare: no support from any organisation for the submitted work; no financial relationships with any organisations that might have an interest in the submitted work in the previous three years, no other relationships or activities that could appear to have influenced the submitted work.

## Authors’ contributions

All authors have contributed significantly to this study. I.L. Meek and H.E. Vonkeman were involved in all phases from the planning of the study to the writing of the manuscript. M.A.F.J. van de Laar was involved in the design of the ACT-CVD database and commented on the statistical analysis of the data, interpretation of the results, and preparation of the manuscript. All authors read and approved the final manuscript.

## Pre-publication history

The pre-publication history for this paper can be accessed here:

http://www.biomedcentral.com/1471-2474/15/174/prepub
